# Annual acknowledgement of reviewers

**DOI:** 10.1186/1752-0509-7-35

**Published:** 2013-04-12

**Authors:** Tim Sands

**Affiliations:** 1 BioMed Central, Floor 6, 236 Gray's Inn Road, London WC1X 8HB, UK

## Abstract

**Contributing reviewers:**

The editors of BMC Systems Biology would like to thank all our reviewers who have contributed to the journal in Volume 6 (2012).

## 

Lipi Acharya

USA

Raj Acharya

USA

Farid Ahmed

USA

Yong-Yeol Ahn

USA

Tatsuya Akutsu

Japan

Réka Albert

USA

Gabriela Alexe

USA

Frank Allgower

Germany

Eivind Almaas

Norway

Jonas Almeida

USA

Miguel Almeida

Spain

Rui Alves

Spain

Gary An

USA

Steve Andrews

USA

Vladimir Anisimov

UK

Maciek Antoniewicz

USA

Ping Ao

China

Elias August

Switzerland

Luc Baeyens

Belgium

Julio Banga

Spain

Debmalya Barh

India

Chris Barnes

UK

Sina-Victoria Barysch

Germany

Josep Bassaganya-Riera

USA

Travis Bayer

UK

Stephanie Bayol

UK

Tara Beattie

Canada

Tim Beissbarth

Germany

Yaakov Benenson

Switzerland

Panayiotis Benos

USA

Frank Bergmann

USA

Corine Bertolotto

France

Katja Bettenbrock

Germany

Sharad Bhartiya

India

Dhruba Bhattacharyya

India

Ginestra Bianconi

USA

Krastan Blagoev

USA

William Blake

USA

Lars Blank

Germany

Joke Blom

Netherlands

Nils Bluthgen

Germany

Gennady Bocharov

Russia

Clive Bowser

UK

Guy Brock

USA

Jean-Luc Bueb

Luxembourg

Paulina Bull

Chile

Anthony Burgard

USA

Peter Buske

Germany

Yudong Cai

China

Laurence Calzone

France

Rodney Camire

USA

Yang Cao

USA

Youfang Cao

USA

Brian Carlson

USA

Javier Carrera

Spain

Jason Carroll

UK

Gunnar Cedersund

Sweden

Christina Chan

USA

Nagasuma Chandra

India

Deepak Chandran

USA

Cheng-Shyong Chang

Taiwan

Daniel Chasman

USA

Xi Chen

USA

Qiong Cheng

USA

Wai Ki Ching

Hong Kong

Byung-Kwan Cho

South Korea

Young-Rae Cho

USA

Seungjin Choi

South Korea

Jong Hyun Choi

South Korea

Sudhir Chowbina

USA

Struck Ciborowski

USA

Mathieu Cloutier

Canada

James Collins

USA

Ana Conesa

Spain

Jürgen Cox

Germany

Travis Craddock

Canada

Peter Csermely

Hungary

Attila Csikasz-Nagy

Italy

Qinghua Cui

China

Qizhi Cui

USA

Bjorn Dahlback

Sweden

Florence D'Alche-Buc

France

Antoine Danchin

France

Stefanie De Bodt

Belgium

Rob De Boer

Netherlands

Luis De Figueiredo

UK

Hidde de Jong

France

Pierre De Meyts

Denmark

Dick de Ridder

Netherlands

Erik De Schutter

Japan

Matthew Delisa

USA

Emek Demir

USA

Youping Deng

USA

Diego Di Bernardo

Italy

Joseph Distefano III

USA

Aris Dokoumetzidis

Greece

Andreas Dräger

Germany

Saliha Durmuş Tekir

Turkey

Omer Dushek

UK

Janusz Dutkowski

Poland

Oliver Ebenhoeh

UK

Birgitta Ebert

Germany

Stephen Edwards

USA

Sol Efroni

Israel

Lukas Endler

UK

Susanne Engelmann

Germany

Roland Ewald

Germany

François Fages

France

Francesco Falciani

UK

Karoline Faust

Belgium

David Fell

UK

Haidong Feng

USA

Michael Fernandez

Canada

Elana Fertig

USA

Jason Flannick

USA

Christian Fleck

Netherlands

Ronan Fleming

Iceland

Virginia Folcik

USA

Stephen Fong

USA

Christian Forst

USA

Jochen Förster

Denmark

Elisa Franco

USA

Jean Marie Francois

France

Thomas Freeman

UK

Leonid Fridlyand

USA

Caroline Friedel

Germany

Cong Fu

USA

Aleksandra Fucic

Croatia

Georg Fuellen

Germany

Akira Funahashi

Japan

Matthias Futschik

Portugal

Gennaro Gambardella

Italy

Olivier Gandrillon

France

Jordi Garcia-Ojalvo

Spain

Krishna Garikipati

USA

Donald Geman

USA

Pierre Geurts

Belgium

Erwin Gianchandani

USA

Stephen Gilmore

UK

Mark Girolami

UK

Paula Gonçalves

Portugal

Yunchen Gong

Canada

Juan Gonzalez

USA

Christopher Gowen

Canada

Eva Grafahrend-Belau

Germany

Ramon Grima

UK

Sergio Grimbs

Germany

Gustavo Gudesblat

Argentina

Justin Guinney

USA

Rudiyanto Gunawan

Switzerland

Xin Guo

USA

Carito Guziolowski

France

William Haldenwang

USA

Jennifer Hallinan

UK

Jing-Dong Han

China

Baris Hancioglu

USA

Anja Hartmann

Germany

Jan Hasenauer

Germany

Ines Heiland

Germany

Matthias Heinemann

Netherlands

Monika Heiner

Germany

Elmar Heinzle

Germany

Steven Henikoff

USA

Christopher Henry

USA

Markus Herrgard

USA

Ralf Herwig

Germany

James Hetherington

UK

Masami Hirai

Japan

William Hlavacek

USA

Thomas Hofer

Germany

Dawei Hong

USA

Andreas Hoppe

Germany

Valerie Hu

USA

Kerwyn Casey Huang

USA

Liang-Tsung Huang

Taiwan

Mike Hucka

USA

Marc-Thorsten Hütt

Germany

C. Anthony Hunt

USA

Taisen Iguchi

Japan

Lucian Ilie

Canada

Elling Jacobsen

Sweden

Dieter Jahn

Germany

Tobias Jahnke

Germany

Neema Jamshidi

USA

Jean-Luc Jannink

USA

Constance Jeffery

USA

Paul Jensen

USA

Jouhyun Jeon

Canada

Matthias Jeschke

Germany

Iain Johnston

UK

Tim Kacprowski

Germany

Lars Kaderali

Germany

Mads Kaern

Canada

Michael Kalafatis

USA

Christoph Kaleta

Germany

Margaret Katz

Australia

Jörn Kekow

Germany

Mustafa Khammash

USA

Dongsup Kim

South Korea

Raimund Kinne

Germany

Justin Kinney

USA

Paul Kirk

UK

Steffen Klamt

Germany

Gunnar Klau

Netherlands

Steen Knudsen

Denmark

Ina Koch

Germany

Michal Komorowski

UK

Dirk Koschützki

Germany

Ekaterina Kotelnikova

Russia

Nicole Krämer

Germany

Andreas Kremling

Germany

Jan Krumsiek

Germany

Yoshihisa Kubota

USA

Vishwesh Kulkarni

USA

Ursula Kummer

Germany

Hiroyuki Kurata

Japan

Isil Kurnaz

Turkey

Christina Kuttler

Germany

Dong-Yup Lee

Singapore

Insuk Lee

South Korea

Sang Hong Lee

Australia

Joungmin Lee

South Korea

Stefan Legewie

Germany

Tanya Leise

USA

Shao Li

China

Zheng Li

USA

Lu Li

UK

Xia Li

China

Jingjing Li

Canada

Xiyan Li

USA

Lei Li

Canada

Jie Liang

USA

Igor Libourel

USA

Wolfram Liebermeister

Germany

Gipsi Lima-Mendez

Belgium

Chung-Yen Lin

Taiwan

Chen-Ching Lin

Taiwan

Jörg Linde

Germany

Wei-Chung Liu

Taiwan

Bo Liu

China

Zhi-Ping Liu

China

Cristina Lo Celso

UK

Ed Louis

UK

Henry Horng-Shing Lu

Taiwan

Zelmina Lubovac Pilav

Sweden

Fabio Luciani

Australia

Camilla Luni

Italy

Huanfei Ma

China

Shisong Ma

USA

Thomas MacCarthy

USA

Aaron Mackey

USA

Paul Magwene

USA

Rodrigo Maillard

USA

Vincenzo Manca

Italy

Susanna Manrubia

Spain

Costas Maranas

USA

Daniel Marbach

USA

Mario Andrea Marchisio

Switzerland

Alberto Marin-Sanguino

Germany

Tim Marler

USA

Tatiana Marquez-Lago

Japan

Diethard Mattanovich

Austria

Aurelien Mazurie

USA

Tommaso Mazza

Italy

Geoff McLachlan

Australia

David McMillen

Canada

Hendrik Mehlhorn

Germany

Sarika Mehra

India

Martin Meier-Schellersheim

USA

Hans Meinhardt

Germany

Eduardo Mendoza

Germany

Aline Metris

UK

Huaiyu Mi

USA

Qi Mi

USA

George Michailidis

USA

Ljubisa Miskovic

Switzerland

Faheem Mitha

USA

Kenji Mizuguchi

Japan

Andrey Molokanov

Russia

Ion Moraru

USA

Sandro Morganella

Italy

Konstantinos Moutselos

Greece

Eric Muller

USA

Yoshiyuki Murata

Japan

Philip Murray

UK

Chris Myers

USA

Manikandan Narayanan

USA

Qing Nie

USA

Jens Nielsen

Sweden

Kay Nieselt

Germany

Zoran Nikoloski

Germany

Kunihiko Nishino

Japan

Yuanling Niu

China

Santosh Noronha

India

Bela Novak

UK

Michael Oldham

USA

Baldo Oliva

Spain

Ana Paula Oliveira

Switzerland

Brett Olivier

Netherlands

Lars Folke Olsen

Denmark

Richard Orton

UK

Diego Oyarzun

UK

Arzucan Ozgur

Turkey

Gultekin Ozsoyoglu

USA

Jürgen Pahle

UK

Debnath Pal

India

Csaba Pal

Hungary

Bernhard Palsson

USA

Chi Nam Ignatius Pang

Australia

Irene Papatheodorou

UK

Jason Papin

USA

Balázs Papp

Hungary

Mark Parcells

USA

Jong Myoung Park

South Korea

Alberto Pascual-Montano

Spain

Kiran Raosaheb Patil

Germany

Andy Perkins

USA

Howard Petrie

USA

Thomas Pfeiffer

USA

Sebastian Polak

Poland

Corrado Priami

Italy

Nathan Price

USA

Carole Proctor

UK

Andrey Ptitsyn

USA

Pere Puigbo

USA

Xiaoning Qian

USA

Ovidiu Radulescu

France

Anu Raghunathan

India

Karthik Raman

India

Giordano Rampioni

Italy

Ravi Rao

USA

Alpan Raval

India

Christopher Rawlings

UK

Jennifer Reed

USA

Markus Rehm

Ireland

Isidore Rigoutsos

USA

Miguel Rocha

Portugal

Juan Rodriguez

Spain

Carlos Rodriguez-Caso

Spain

Maria Rodriguez-Fernandez

USA

Stefano Rovetta

Italy

Jianhua Ruan

USA

Andreas Ruepp

Germany

Ann Rundell

USA

Eytan Ruppin

Israel

Achuthsankar Nair

India

Ahmet Sacan

USA

Julio Saez-Rodriguez

UK

Supreet Saini

India

Shigeru Saito

Japan

Werner Sandmann

Germany

Moises Santillan

Mexico

Roschen Sasikumar

India

Jeffrey Saucerman

USA

Uwe Sauer

Switzerland

Tim Sauer

USA

Carlos Saura

Spain

Herbert Sauro

USA

Arnold Saxton

USA

Andrea Sboner

USA

André Scherag

Germany

Thomas Schlitt

UK

Falk Schreiber

Germany

Hildgund Schrempf

Germany

Martin Schuster

USA

Joachim Selbig

Germany

Junhee Seok

USA

Yaki Setty

Germany

Harish Shankaran

USA

Shayan Sharif

Canada

Yao Shen

USA

Ronglai Shen

USA

Motoki Shiga

Japan

Yu Shyr

USA

Marco Silano

Italy

Evangelos Simeonidis

Luxembourg

Gertien Smits

Netherlands

Jacky Snoep

South Africa

Joshua Socolar

USA

Sylvain Soliman

France

Jiuzhou Song

USA

Hermona Soreq

Israel

Ganesh Sriram

USA

Guy-Bart Stan

UK

Jörg Stelling

Switzerland

Paul Sternberg

USA

Chris Stevenson

UK

Gautier Stoll

France

Gustavo Stolovitzky

USA

Ronny Straube

Germany

Sean Stromberg

USA

Jörg Stülke

Germany

Traian Sulea

Canada

Jingchun Sun

USA

Patrick Suthers

USA

Arthur Tenenhaus

France

Andrew Teschendorff

UK

Douglas Thamm

USA

Elizabeth Theil

USA

Johan Thevelein

Belgium

Denis Thieffry

France

Ines Thiele

Iceland

Chabane Tibiche

Canada

Tina Toni

USA

Cong Trinh

USA

Huai-Kuang Tsai

Taiwan

Krasimira Tsaneva-Atanasova

UK

Koji Tsuda

Japan

Adelinde Uhrmacher

Germany

Korkut Uygun

USA

Federico Vaggi

Italy

Kaspar Valgepea

Estonia

Aalt-Jan Van Dijk

Netherlands

Marco Vanoni

Italy

Wynand Verwoerd

New Zealand

Susana Vinga

Portugal

Ganesh Viswanathan

India

Yoram Vodovotz

USA

Jiri Vohradsky

Czech Republic

Dagmar Waltemath

Germany

Dirk Walther

Germany

Yong Wang

China

Rui-Sheng Wang

USA

Edwin Wang

Canada

Liming Wang

USA

Xuewei Wang

USA

Junwen Wang

China

Ruiqi Wang

China

Jin Wang

USA

Guanyu Wang

USA

Louis Wehenkel

Belgium

Zhi Wei

USA

Johan Westerhuis

Netherlands

Wolfgang Wiechert

Germany

Christoph Wierling

Germany

H Steven Wiley

USA

Ned Wingreen

USA

Michael Wink

Germany

Carsten Wiuf

Denmark

Jana Wolf

Germany

Johannes Wollbold

Germany

Fang-Xiang Wu

Canada

Wei-Sheng Wu

Taiwan

Xiaogang Wu

USA

Stefan Wuchty

USA

Ioannis Xenarios

Switzerland

Jianhua Xing

USA

Yoshihiro Yamanishi

Japan

Guiying Yan

China

Byung-Jun Yoon

USA

Akihiko Yoshimura

Japan

Ronen Zaidel-Bar

Singapore

Mattia Zampieri

Italy

Tao Zeng

China

Weiwen Zhang

China

Zhaolei Zhang

Canada

Tongli Zhang

UK

Da-Peng Zhang

China

Xiaomiao Zhao

China

Xingming Zhao

China

Deming Zhao

China

Deyou Zheng

USA

Tianshou Zhou

China

Shuigeng Zhou

China

Zhihao Zhuang

USA

Anna Zhukova

France

Pietro Zoppoli

Italy

Xiufen Zou

China

